# Multimodal Warnings Design for In-Vehicle Robots under Driving Safety Scenarios

**DOI:** 10.3390/s23010156

**Published:** 2022-12-23

**Authors:** Jianmin Wang, Chengji Wang, Yujia Liu, Tianyang Yue, Yuxi Wang, Fang You

**Affiliations:** 1 Car Interaction Design Lab, College of Arts and Media, Tongji University, Shanghai 201804, China; 2 Shenzhen Research Institute, Sun Yat-Sen University, Shenzhen 518057, China; 3 Nanchang Research Institute, Sun Yat-Sen University, Nanchang 330224, China

**Keywords:** multimodal warnings, interaction design, transparency, in-vehicle robots

## Abstract

In case of dangerous driving, the in-vehicle robot can provide multimodal warnings to help the driver correct the wrong operation, so the impact of the warning signal itself on driving safety needs to be reduced. This study investigates the design of multimodal warnings for in-vehicle robots under driving safety warning scenarios. Based on transparency theory, this study addressed the content and timing of visual and auditory modality warning outputs and discussed the effects of different robot speech and facial expressions on driving safety. Two rounds of experiments were conducted on a driving simulator to collect vehicle data, subjective data, and behavioral data. The results showed that driving safety and workload were optimal when the robot was designed to use negative expressions for the visual modality during the comprehension (SAT 2) phase and speech at a rate of 345 words/minute for the auditory modality during the comprehension (SAT 2) and prediction (SAT 3) phases. The design guideline obtained from the study provides a reference for the interaction design of driver assistance systems with robots as the interface.

## 1. Introduction

**Figure 1:**
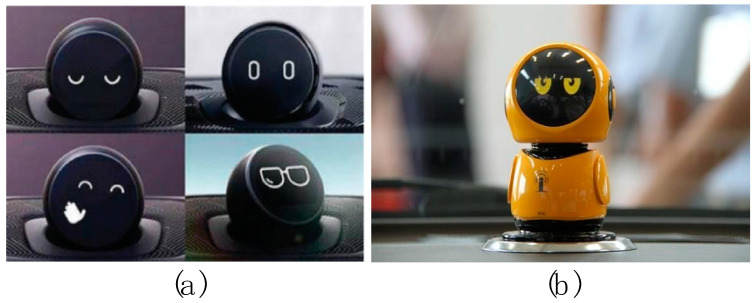
(**a**) NIO’s NOMI. (**b**) BYD’s Qin.

**Figure 2:**
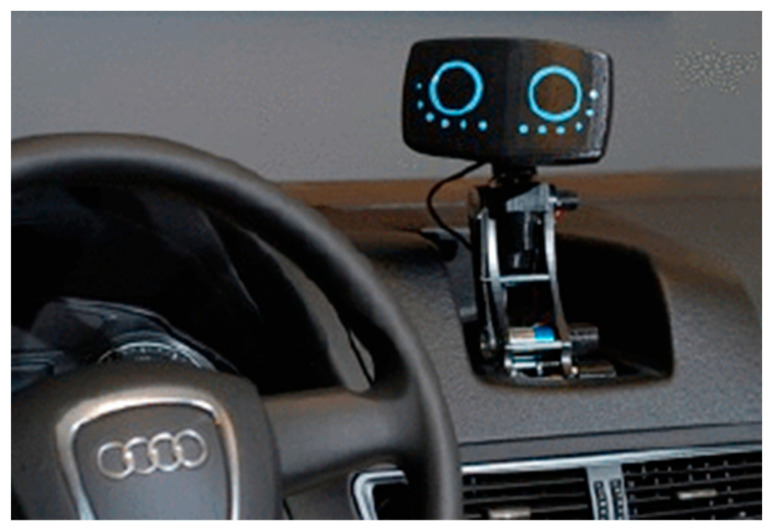
Kenton William ‘s AIDA.

Driving safety is critical for vehicle drivers and other road users (e.g., pedestrians, cyclists, bicyclists, motorcycles, etc.). Human factors play a significant role in automotive safety. According to a survey conducted by the National Highway Traffic Safety Administration (NHTSA), human-caused incidents account for 94% of all vehicle crashes [1]. Today, cars are equipped with a variety of driver assistance systems to help drivers drive more safely. These driver assistance systems have become one of the most active areas of Intelligent Traffic System (ITS) research [2,3]. In recent years, with the development of automation technology, natural language processing, and emotional computing, many intelligent assistive systems have been equipped with anthropomorphic robotic bodies. Driver assistance systems have evolved from human–machine interaction to human–robot interaction (Figure 1), and automakers have begun using robots as the interface of driver assistance systems. The research of Williams et al. showed that dynamic robots (Figure 2) had a significant impact on reducing the user’s cognitive load and distractions [4]. These robots are generally anthropomorphic and, thus, more like human passengers, which enhances the driver’s concern for safe driving [5,6,7]. Since robots generally have displays and speakers, vehicle-mounted robots can provide multimodal warnings with combined visual modality and auditory modality by voice and expressions. Outputting facial expressions and speech is a fundamental capability of in-vehicle robots. Several studies have shown that robot facial expressions and speech have additional positive effects on driving safety [8,9]. Many studies have shown that multimodal warnings in cars are more beneficial for driving safety than unimodal warnings [10,11], both for manual [11,12] and highly automated [13] vehicles.

However, improperly designed multimodal warnings for in-vehicle robots can affect driving safety. Therefore, designers need design guidelines to reduce dangerous multimodal warnings. The main reason for safety risks is the limitation of the driver’s cognitive load on each modality of the robot. It is estimated that up to 95% of the information received while driving is visually recognized [14], so visual warnings may have to compete for the visual resources needed for vehicle control and may be distracted by a secondary visual task in the vehicle [15]. In addition, a sudden auditory warning signal may startle the driver and trigger distraction, creating a safety hazard [16]. Poorly designed multimodal warnings may lead to slower responses or increased error rates [17] and may cause a potentially relevant deleterious cross-modal effect [18,19]. In the current study, multimodal warnings are mostly triggered simultaneously, and there is a lack of studies on the sequential display of each modality. The content of the warnings provided by each modality is also not clearly defined.

In-vehicle robots should contribute to driving safety. Thus, how to ensure that robot warnings do not compromise driving safety in a human–robot co-driving scenario is an important interaction design issue. The research on multimodal warning design for in-vehicle robots, especially the multimodal warning with combined visual modality and auditory modality, is of critical importance for human–robot driving safety during human–robot co-driving. This includes when each modal warning is displayed, what is included in each modal warning, and how robots express their facial expression and give voice warnings. This paper first discusses the content and coordination of robot expressions and speech based on SAT theory, then discusses robot facial-expression valence and speech rate. Then, different multimodal designs are experimentally evaluated using a robot equipped with a facial screen and a speaker in a scenario where the driver makes a mistake (speeding). The experimental results were compared to draw conclusions about the multimodal warning design of the robot as an interactive interface for driver assistance systems. This research provides design guidelines for the multimodal warnings provided by in-vehicle robots to help drivers drive more safely with reducing the impact of the warnings themselves on driving safety.

This paper first introduces the theory of transparency for human–robot communication in Section 2 and presents related works on visual warnings and auditory warnings of in-vehicle robots. Then, in Section 3, we introduce the design of the multimodal warning for robots based on SAT theory and perform SAT mode hypothesis. Section 4 describes our two experiments on our in-vehicle robot in a driving simulator and the dependent variable (car data and human factors). Section 5 presents the results of the experiments on each evaluation dimension, which show that there is a robot SAT mode, speech rates, and facial-expression valence that are more conducive to driving safety. Finally, Section 6 discusses the findings of this study on multimodal warnings design for in-vehicle robots under driving safety scenarios, suggestions for human–robot design, and future research directions.

## 2. Related Works

### 2.1. Robot Transparency during Human–Robot Interaction

To correct a driver’s driving errors or to provide other driving advice proactively, the robot should make reasonable decisions based on the information of the driver and the external environment, and output at least the results to the driver through warning signals. However, the output of the robot should not only be the result of its decision but also provide some context of the decision. The autonomous robot is an intelligent agent. Understanding the purpose and behavior of the agent is important for proper human understanding and sound judgment of the agent [20]. Van Dongen et al. [21] pointed out that participants’ awareness of the agent’s decision reasoning process influences their reliance on the agent. This understanding of the agent is referred to as “transparency”.

Transparency is suitable for studying the timing and content of warning signal outputs. It is a continuous series of processes by which a human understands the purpose and behavior of an agent based on the information provided by the agent and contains the context of the final output. To sustain the effectiveness of robot warnings, the transparency of robots must be improved. Transparency can be enhanced by conveying clearer information. Lee [22] recommended that system designers make the 3Ps (purpose, process, and performance) of the system and its history available to the operator in order to optimize the transparency of automation to the operator. Chen et al. proposed a situational awareness-based agent transparency theory (SAT) [23] to explain what information contributes to transparency. The SAT model is based on Endsley’s Situational Awareness (SA) theory [24], which analyzes the SA level of an agent to explain what information contributes to transparency. Situation awareness-based agent transparency theory defines agent transparency as a descriptive quality of an interface, where the operator understands the intention, reasoning process, and future plans of an intelligent agent.

Situational Awareness (SA) theory [24] proposes three levels, including SA Level 1, the perception of elements in the environment; SA Level 2, the understanding of these elements; and SA Level 3, the prediction of their state in the near future. The SAT model is based on SA theory and includes three levels that describe the content provided by the agent to the driver in order to maintain transparency between the agent and the human. SAT1 level is the perception level, whose content is the agent’s goal and its perception of the environment state. SAT2 level is the prediction level, whose content is the agent’s understanding of the situation and the reasoning process of the action. SAT3 level is the prediction level, whose content corresponds to the agent’s prediction of the future outcome.

The three levels of SAT can be considered consecutive periods when the robot provides warnings and contains different information. The SAT level of warnings from different modalities should provide the driver with the appropriate level of transparency to enhance driving safety

### 2.2. In-Vehicle Robot’s Visual Warnings

Vision is the primary access to information for drivers while driving. A study has suggested that up to 95% of the information received while driving is visually recognized [14]. Traditional visual warnings mostly display warning messages through changes in the interior lights of the vehicle. Typically, the driver is alerted by flickering LEDs in the interior rear-view mirror [25], or the changing background color, signal word, and pulse rate on a monitor [26]. The robot mainly transmits visual signals through the screen.

Robots with screens can transmit visual signals by displaying symbols, colors, etc. Unlike general visual interaction interfaces such as flashlights, robots need to maintain anthropomorphism and, therefore, need to maintain their facial display most of the time. The facial visual signals of a robot can convey emotions through expressions and can indicate the intention to interact with and attract the attention of the driver through head and eye gaze behaviors [27]. In the design of facial expressions for robots, Young [28] et al. used simulated and exaggerated facial expressions and hand gestures to provide powerful expressive mechanisms for robots.

The current study focused on the impact of facial-expression valence on warning effects and driving performance. Expression valence has been demonstrated to influence individuals’ emotional processing, with negative expressions being more likely to be detected and recognized [29] and to activate the brain [30]. Kern et al. and Talmi et al. discovered that when attention resources are scarce, attention and memory for positive and neutral images drop [31,32]. Driving settings require a significant amount of attentional resources, which may amplify this expression valence bias [33]. Thus, the in-vehicle robot’s facial expression valence can be chosen to increase the attractiveness of visual warning signals to drivers without risking their safe driving.

### 2.3. In-Vehicle Robot’s Auditory Warnings

The robot’s voice is an auditory warning signal to the driver. Drivers often respond faster to audio signals than to visual warnings [34], which is highly relevant to driving safety. More crucially, audio stimuli are perceived non-directionally; drivers hear auditory stimuli regardless of where their attention is oriented [35]. That makes auditory a suitable way to convey warning signals. Graham designed auditory icons played by speakers for an in-vehicle collision avoidance application [35], and Otto Carlander played 3D audio in headphones [36].

The auditory signals provided by the robot are mostly verbal speech signals played by speakers. This speech-based interface that incorporates textual content information has been demonstrated to alleviate driver fatigue [37] and operate as a mediator of human emotion [38]. Eriksson and Stanton hypothesized that verbal speech signals used in engagement with robots might also help bridge the ‘gulf of evaluation’ between humans and machines [39]. According to Forster et al., verbal speech has a number of advantages over generic auditory output in terms of agent trust, anthropomorphism, usability, and acceptance during human–agent contact [40]. The NHTSA provides a design guideline for in-vehicle voice message warnings, which stipulate speech quality, gender, the number of discourse units, and speech rate [41].

This paper explores the key factors that influence the audio warnings of robots. E. C. Haas et al. [42] proposed to design auditory warning signals with three parameters assigned, speed, pitch, and volume. Many studies have considered perceived urgency as a key factor in auditory warning signals and have explored the different effects of acoustic and non-acoustic parameters [43]. Neuhoff, J. G. et al. [44] found that a perceptual interaction between pitch and loudness between dynamic changes in pitch and loudness. Baldwin et al., in their study of how loudness interacts with semantics, found that loudness alone did not significantly impact ratings of perceived urgency. A study by Ofuji and Ogasawara [45] showed that urgency could be influenced by the speed factor alone by up to 39%. In this study, the synthetic speech rates of different robots were compared to find the appropriate robot voice channel performance.

## 3. Design Methods

Previous studies on multimodal warnings have proposed some design guidelines [17,46]. First, outputs from different modalities must be in close temporal proximity (temporal rule). Second, the larger the effects, the closer the stimuli are presented in space (spatial rule). Third, the strength of the stimuli provided has an inverse relationship with the amplitude of the multisensory effect (principle of inverse effectiveness). However, the multimodal warnings discussed in these studies are mostly transient and are used to attract the driver’s attention in an instant or to direct the driver’s attention to a specific location. Besides these transient warnings, the robot can also deliver more complex warnings with more content. This type of warning signal has length, e.g., the robot can warn the driver of speeding and inform the driver of the location of the speed radar ahead. Therefore, new design guidelines are needed for multimodal warnings of in-vehicle robots.

**Figure 3:**
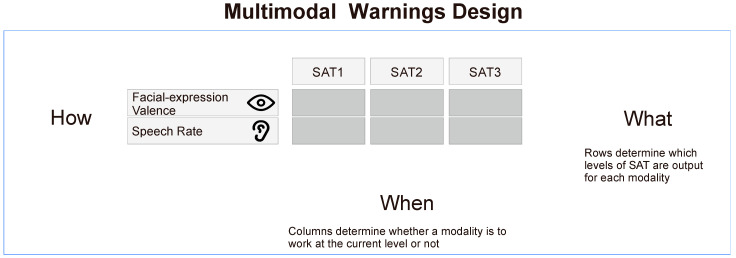
Design of “when”, “what”, and “how” of multimodal warnings of an in-vehicle robot.

The design robots’ multimodal warnings involves the constraint of the timing (when), the content (what), and the performance (how) of the warning signals from each modality (Figure 3). “When” includes the order and timing of each modality’s warning signals. Each channel should provide information in an appropriate time, not occupy the attention resources, and achieve better cooperation. “What” includes the content and amount of information that each modality needs to convey. The amount of content provided by each modality should help drivers understand the information and enhance transparency. “How” includes the performance of the visual and auditory modalities in conveying information, and this study focuses on the speech rate and the robot’s facial-expressions valence.

Based on SAT theory, this paper investigates the “content” of multimodal warnings through the robot’s transparency and the “timing” of warnings through the robot’s successive SA phases. SAT theory assumes that the robot needs to provide more than just outcome information to the driver to maintain proper transparency. The theory provides three successive phases based on situational awareness theory and specifies what each phase entails. The transparency of the robot can be expressed through multiple modalities, so the time period in each modality works and what it expresses can be chosen. As shown in Figure 3, this study built a multimodal warning design model for the robot based on the SAT model of the robot. The core of the model is the table in the middle of the figure. The column header represents the three SAT levels, and the row headers are the visual modality and the auditory modality. The horizontal arrows in the table point to “what” because each row of the table represents the three SAT levels of information that each modality can provide. It is used to design the amount of information contained in each modality, which represents the “what” of multimodal warnings. The vertical arrows in the table point to “when” because each column represents the information that can be output in either the auditory or visual modality during a specific SAT phase. It is used to design which modalities to perform at each time period, which determines the “when” for multimodal warnings. Filling in the table constrains both the “what” and “when” of the multimodal warnings and determines the SAT mode of the robot by designing the modality to work at specific times. The “how” at the left end of the row headers indicate that the expression of each modality also needs to be designed.

**Figure 4:**
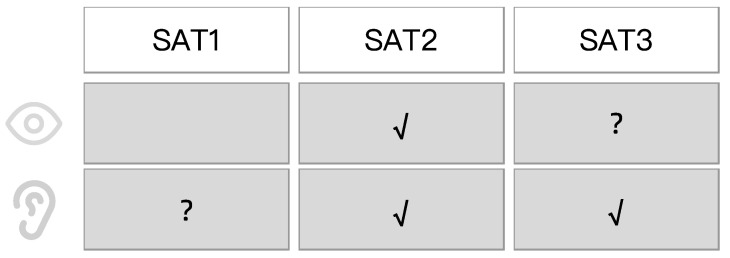
SAT model assumptions for multimodal warnings.

This study did not exhaust all SAT modes. In order to convey the basic information of the warning to the driver, the warning should at least include explicit SAT2-level information (understanding and informing the driver of the current state) and SAT3-level information (predicting and informing the driver of what they should do then), which need be delivered through the robot voice. This is also consistent with previous research showing that an intelligent robot generally needs to have SAT2 understanding and SAT3 prediction [43]. The visual channel does not need to convey SAT1-level information because the robot’s default expression indicates that the robot is in perception. Therefore, SAT2-level visual signals need to be provided to differentiate from SAT1-level robot states. Considered together, the possible SAT modes for robot multimodal warnings in driving safety warning scenarios are given in Figure 4. In the table, “?” is the information needed to verify whether the current level is provided through the current channel through experiments.

For “how” of the in-vehicle robot’s multimodal warnings design, we investigated the robot’s facial-expression valence and speech rate. The in-vehicle robot we used had a screen and a speaker to output facial expressions and voice. In this study, we conduct an experimental comparison of the SAT mode assumptions described above, two facial-expression valences, and four speech rates to determine what multimodal warnings designs are more advantageous to driving safety.

## 4. Simulation Experiment

### 4.1. Experimental Design

**Figure 5:**
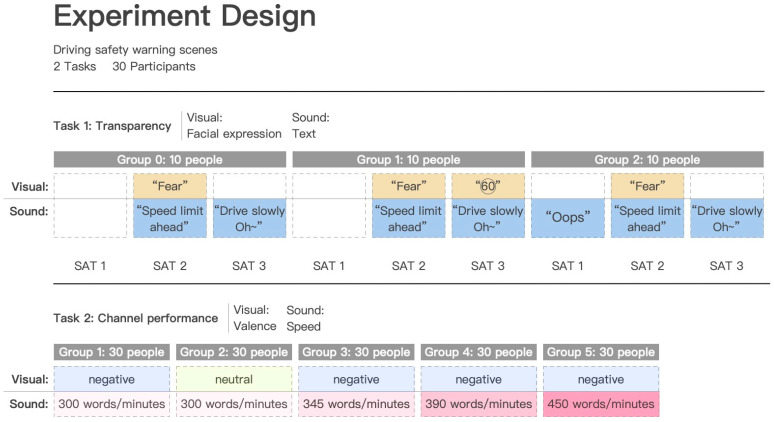
Different transparency modes for task 1; different speech, speed, and facial-expression valence for task 2.

The study conducted two rounds of experiments using the same scenario (Figure 5). Experiment 1 was a between-subjects experiment in which three experimental groups were designed according to the three possible transparency modes included in Figure 4, and each subject was assigned to one of the three experimental groups according to the Latin square and participated in the driving task only once. Experiment 2 was a within-subjects experiment with five experimental groups. Among them, Experimental Group 1 was the standard experimental group, Experimental Group 2 was the visual modality control group, and Experimental Groups 3, 4, and 5 were the auditory modality control group; each subject had to participate in all five driving tasks, and the order of participation in the tasks was determined by the Latin square equilibrium order effect. The two rounds of the experiment are shown in the figure. In each driving task, subjects were asked to familiarize themselves with the driving simulator until they could drive smoothly in the left lane and maintain a speed of about 30 km/h. An example interaction scenario was used to ensure that the driver could interact with the robot properly, thus creating a certain familiarity with the robot and deepening the subject’s trust in it.

We designed speeding reminders for drivers as a typical scenario of driving safety warning scenarios. The lead experimenter introduced the purpose of the study to the subjects, which was to test a newly developed in-vehicle robot while driving. The simulator’s driving environment was a two-way four-lane highway with a 60 km/h speed limit. The main experimenter’s description of the scenario was that the subject was going to pick up someone at the airport, the plane was about to land, and the driver need to increase the speed of the car to above 70 km/h. So, the driver was required to drive in the left lane, accelerate to 70 km per hour as described by the lead experimenter, and then slow down to 60 km per hour when the robot notified him. Once the speed was above 60 km/h, the robot took the initiative to remind the driver of the speed limit.

#### 4.1.1. Experiment One

Experiment 1 was a between-subjects experiment. The experimental variable were SAT modes of the robot’s multimodal warnings. Based on the transparency assumption of Section 3 (Figure 4), three schemes with different SAT modes are designed. The SAT mode of scheme 1 (Group 0) is that the robot outputs both speech and facial expressions at the SAT2 level and speech at the SAT3 level. The SAT2-level expression is the negative expression “Fear” because the robot understands that the driver is speeding and is afraid of him. The SAT2-level and SAT3-level expressions are “Speed limit ahead, you are speeding” and “Drive slowly Oh~”, respectively. Scheme 2 (Group 1) adds the SAT3-level expression to Scheme 1, showing the driver the speed to be slowed down. Scheme 3 (Group 2) adds the SAT1-level expression “Oops!” to Scheme 1. Group 1 and Group 2 compare the need for SAT1-level visual information and SAT3-level auditory information, respectively.

To convey a complete warning to the driver, each experimental group delivered SAT2 information (so that the driver understands the speed limit ahead) and SAT3-level information (predicting the slowing behavior the driver should make) via robot speech. This is also consistent with previous research showing that an intelligent robot generally needs to have SAT2 understanding and SAT3 prediction [47]. No group with SAT1 visual modality warning was set up because the robot was always performing awareness when the driver was not actively interacting with the robot. Groups 1 and 2 contrasted the need to provide visual information for SAT1 level and auditory information for SAT3 level, respectively.

#### 4.1.2. Experiment Two

Experiment 2 was a within-subjects experiment. The experimental variable for the five schemes was the speech rate and facial-expression valence of the robot’s multimodal warnings. Based on the SAT mode of Scheme 1 in Experiment 1, Experiment 2 contained five experimental groups. The expression potency of the robot in Experimental Group 1 was negative, and the speech rate was 300 words/minute. Experimental group 2 compared the expression potency of the robot. The facial-expression valence of Experimental Group 2 was neutral. Experimental Groups 3, 4, and 5 compared the robot’s speech rate of 345 words/minute, 390 words/minute, and 450 words/minute, respectively.

We synthesized the robot’s voice based on the NHTSA design guidelines for voice message warnings during driving [48]. The synthesized voice was a female voice because female voices convey a sense of urgency more easily than male voices. We used three linguistic information units to organize the discourse, specifically: “There is a speeding camera 500 m ahead”, “The speed limit is 60” and “You have exceeded the speed limit”. The three units indicate the distance of the speeding camera ahead, the speed limit, and the driver’s speeding behavior, respectively. The English speech is based on 150 wpm, and three speech speeds are set up based on the urgency of the warning, which are 1×, 1.15× and 1.3× 150 wpm, respectively. Based on this, we set our speech speed control in the same way, choosing the current average speech speed of CCTV news broadcast of 300 words/min [49] as the baseline speech speed, setting up three speech speeds of 1×, 1.15×, and 1.3×, and adding an additional 1.5× speech speed as the limit speech speed for the exploration, i.e., 300 words/min for Experimental Group 1, 345 words/min for Experimental Group 3, 390 words/min for Experimental Group 4 and 450 words/min for Experimental Group 5. To eliminate the effect of speech loudness on driving performance, the loudness of all voice samples was equally set to 70 dB.

We set two expressions for the robot, negative (FEAR) and neutral (MILDNESS). The reason for not setting positive expressions is that the robot should not show positive emotions in this scene where the driver is driving dangerously (speeding). Negative expressions originate from the robot frightened of dangerous driving behavior, where the robot is more like a passenger in the car driving together with the driver and will be worried about the driver and its own safety; the robot with neutral expressions is more like a driving assistant, reminding the driver with a more objective and calm attitude. These two expressions are of the same length.

### 4.2. Experimental Subjects

Thirty Chinese participants, i.e., twenty-five males and five females (ages ranged from 22 to 40 years, *M* = 28.2, *SD* = 5.83, indicating that their ages were primarily between 22 and 34), were recruited via questionnaires that included demographic information about the participants, 83.8 percent of whom had a university or higher education (*n* = 25), followed by junior college (13.3 percent, *n* = 4), and high school education or less (3.3 percent, *n* = 1). They all drove more than twice or three times per week, 70% (*n* = 21) drove electric vehicles, and 80% (*n* = 24) were familiar with in-vehicle robots. Prior to data collection, each subject provided informed consent. The participants had normal eyesight, either uncorrected or corrected, normal color vision, normal hearing in both ears, and no history of mental illness.

### 4.3. Experimental Environment

#### 4.3.1. Driving Simulation

**Figure 6:**
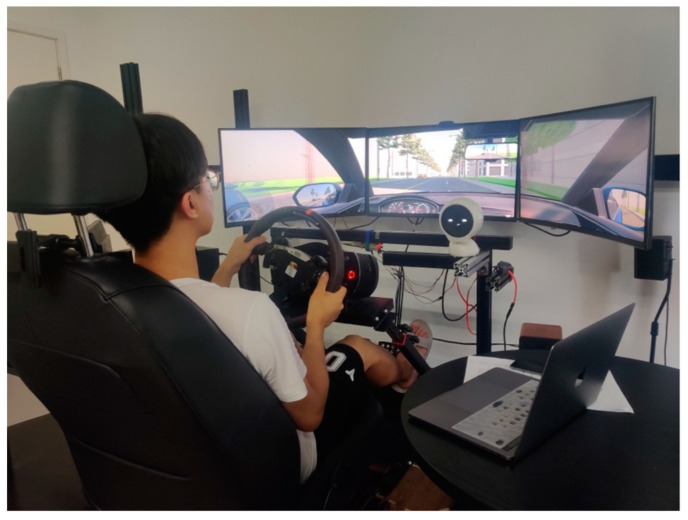
Driving simulation.

The driving simulator (Figure 6) and monitoring system used in this study was independently developed based on Unity. The console contains all the necessary driving components, including the seat, fanatec steering wheel and pedals, as well as a full set of a virtual dashboard and rearview mirrors. In front of the simulator are three large monitors that provide an image of the road environment. The simulator is connected to a Sony stereo playing simulated car engine sounds (±25 dB). There is no other noise in the test area. The study was conducted in the Carlxd lab at the School of Arts and Communication, Tongji University. 

#### 4.3.2. In-Vehicle Robot

**Figure 7:**
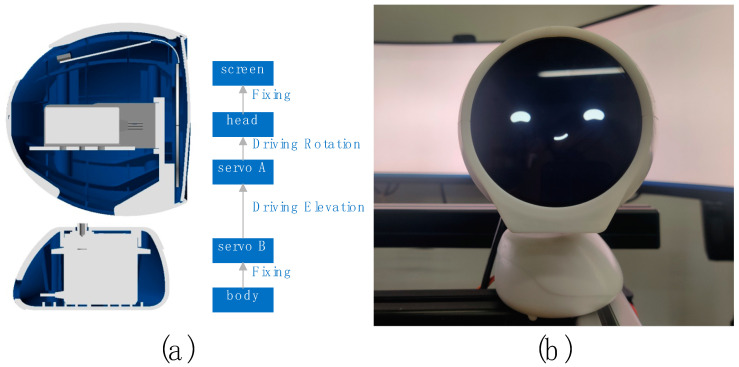
(**a**) Structure of “XiaoV”. (**b**) Appearance of “XiaoV”.

**Figure 8:**
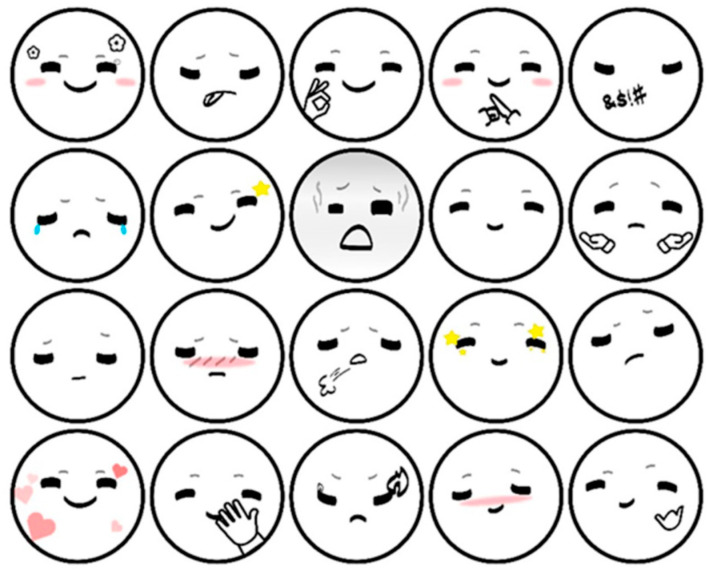
XiaoV’s facial expression set.

The robot system was developed based on Arduino [50] and Unity [51], consisting of a small robot called “XiaoV” with a two-degree freedom platform (Figure 7a), a screen and a speaker (Figure 7b), and a tablet computer running the control program. The control program is used to drive the robot’s pre-programmed behavior, which is triggered by virtual buttons on the tablet’s screen. In this experiment, the key behaviors of the robot are triggered by the operator when the vehicle speed is monitored up to 79 km/h. The other performances of the robot are subsequently triggered according to the driver’s behavior. We designed a series of facial expressions for the robot (Figure 8). Each expression can express the emotion to be expressed well.

The interactive interface of the robot involves both visual and auditory interfaces. The visual interface is displayed on the upper part of the robot through a 3.4” screen, featuring the robot’s facial expressions, with a black background on the face, white features, and colorful auxiliary graphics. The auditory interface uses the Chinese language, using the speech synthesis webAPI provided by the open platform of KU XUNFE for speech synthesis, and the pronouncer chose a female voice, “Yifei”.

### 4.4. Dependent Variable

The dependent variables include vehicle data and driver sweep data recorded through the simulator, and human outcomes obtained through subjective scales. Each subject was asked to fill in an online questionnaire after the experiment. The content of the online questionnaire was subjective scales.

#### 4.4.1. Car Data

The vehicle data is collected by the driving simulator continuously at a rate of 14–20 data points per second, which can be processed to produce a range of vehicle speed marks and left lane distance marks during the interaction phase. Due to the highly unpredictable nature of the car speed in this scenario, the speed marks are utilized only as a reference. The left lane distance is the distance between the driven car and the left lane line; therefore, the standard deviation of the left lane distance can be used to analyze the lane deviation, which indicates the driver’s lateral control ability and can directly show the vehicle’s driving safety condition.

#### 4.4.2. Trust

Lee et al. suggested that trust and transparency are essential components of human–automation cooperation [52]. It can be considered as an attitude from cognitive information (i.e., views about an object), affective information (i.e., feelings and emotions towards an object), and behavioral information (i.e., past or present interaction with an object) [53]. Assessing trust helps with understanding the driver’s acceptance of the robot and the collaboration between human–robot teams [54]. Muir [55] proposed a trust-based model for vehicle automation that includes three trust dimensions: predictability, dependability, and loyalty. The current experiment assesses trust across the three dimensions. The scale options were set from 1 to 7, with higher scores representing a higher degree.

#### 4.4.3. Workload

Workload is a multidimensional concept that refers to a person’s psychological stress or information processing capability when executing a task that involves mental stress, time constraints, task difficulty, operator ability, and effort level, among other things [56]. Workload can greatly affect the performance of human–robot teams. The workload of interacting with the robot can increase or decrease the driver’s driving workload. In this study, workload was assessed using the Driving Activity Load Index (DALI), which measures workload in the attention, visual demand, auditory demand, time demand, distractions, and situational stress dimensions. Scale options were set according to 1 to 10, with higher scores representing a correspondingly higher degree.

#### 4.4.4. Usability

Usability is an essential quality that needs to be possessed by artifacts in general and can describe the quality of appropriateness to a purpose of any particular artifact well [57]. If usability is questioned, especially for a robot with which people are unfamiliar, people are likely to stop using it. Usability testing of robots allows analysis and evaluation of the impact of robot design on the driving experience. There are different ways of measuring usability. The usability questionnaire used in this study was the ASQ (After-Scenario Questionnaire) [58] questionnaire, which is a standard questionnaire for evaluating usability based on the whole task. It has the benefit of evaluating three projects: Ease of Task Completion, Time Required to Complete Tasks, and Satisfaction with Support Information, which can be used in similar usability studies. Participants were required to measure usability for that task after each completion. Scale options were set according to 1 to 7, with higher scores representing a correspondingly higher degree.

#### 4.4.5. Sweeping

We refer to the driver’s behavior of taking the visual focus away from the road ahead as sweeping. Drivers can ignore the lane because of their interest in the performance of a physical robot, which can be dangerous, so the analysis of sweeping glances may reveal negative effects of robots on driving safety. NHTSA states that the average sweep time should not exceed 2 s [48]. A single sweep length exceeding 2 s is also perceived as a risky distracting behavior [59]. Our observations for sweeps were extracted from videos recorded by the simulator. The data contained the number of sweeps in each SAT level as well as the single sweep duration. The total number of sweeps and the total sweep duration of the robot were also calculated.

## 5. Results

A total of 30 subjects participated in the experiment, and Car data, Trust, Workload, Usability, and Sweeping data were collected from each subject. Experiment 1 was a between-subjects experiment with three experimental groups, and each subject participated in one experimental group, so the sample size for each experimental group was 10. Experiment 2 was a within-subjects experiment, so the sample size for each experimental group was 30.

A two-tailed significance level of 0.05 and was used for all tests. One-way ANOVA was used for each index to test for significant differences, and multiple comparisons were used in post hoc tests to test for differences between experimental groups. The experimental evaluation refers to several dimensions from the dependent variable in Section 4.4 above. No single sweep of more than 2 s was found in the sweep data. Therefore, only the total sweep time and number of sweeps are discussed.

### 5.1. Experiment One

#### 5.1.1. Safety

**Figure 9:**
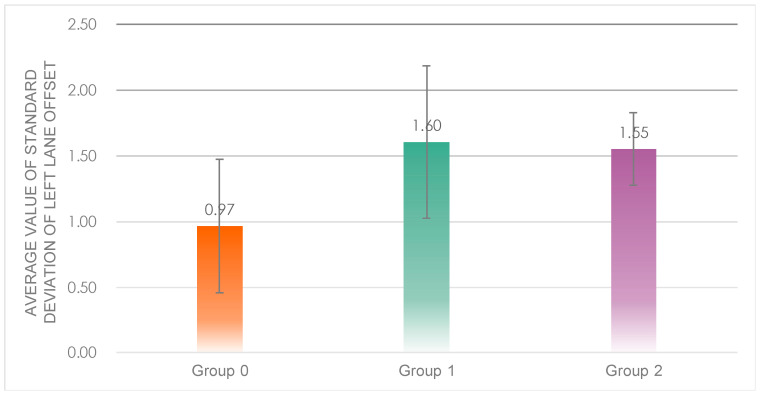
Average value of standard deviation of left lane offset of the three groups.

A one-way ANOVA was performed on the data from the three groups (Figure 9), whose results showed that there was a highly considerable difference in the average value of standard deviation of left lane offset between the three groups (*F*(2, 32) = 6.906, *p* = 0.00 < 0.05). Post hoc tests revealed that the standard deviation of lane shift was significantly lower in Group 0 (*M* = 0.97, *SD* = 0.51) than in Group 1 (*M* = 1.60, *SD* = 0.58) and Group 2 (*M* = 1.55, *SD* = 0.28), with no significant difference between Group 1 and Group 2.

From the dimension of vehicle safety, it was considered that Group 0, which had a significantly better level of lateral vehicle control, was safer.

#### 5.1.2. Workload

**Figure 10:**
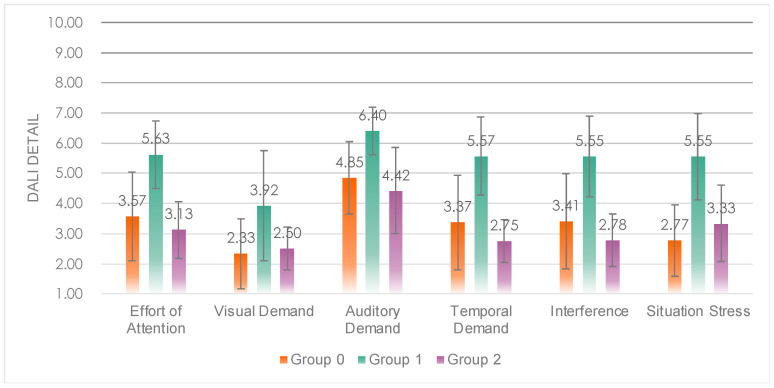
Detail values of the DALI scale of the three groups.

Multiple comparison analysis revealed that Group 1 had a significantly higher workload (Figure 10) than Group 0 (*p* = 0.000 < 0.05) and Group 2 (*p* = 0.002 < 0.05), while Group 0 and Group 2 did not show significant differences in average workload and details. This indicates that the presence of SAT3-level visual information in Group 1 led to a significant increase in workload, while the presence or absence of SAT1-level auditory information (“Oops”) did not significantly affect workload.

#### 5.1.3. Usability

From the average value of usability scores, it can be concluded that there is no significant difference between the three groups (*F*(2, 56) = 0.284, *p* = 0.754). In the usability score details, there were also no significant differences in the three dimensions of satisfaction with difficulty (*F*(2, 51) = 1.471, *p* = 0.239), satisfaction with time spent (*F*(2, 49) = 0.725, *p* = 0.489), and satisfaction with help information (*F*(2, 56) = 0.310, *p* = 0.735).

#### 5.1.4. Trust

From the average trust score, it can be concluded that there is no significant difference between the three groups (*F*(2, 56) = 0.311, *p* = 0.734). The details of the trust score showed that there was also no significant difference above the dimensions of predictability (*F*(2, 52) = 0.022, *p* = 0.978), dependability (*F*(2, 52) = 0.123, *p* = 0.885), and desire to continue using (*F*(2, 49) = 0.159, *p* = 0.854).

#### 5.1.5. Sweeping

**Figure 11:**
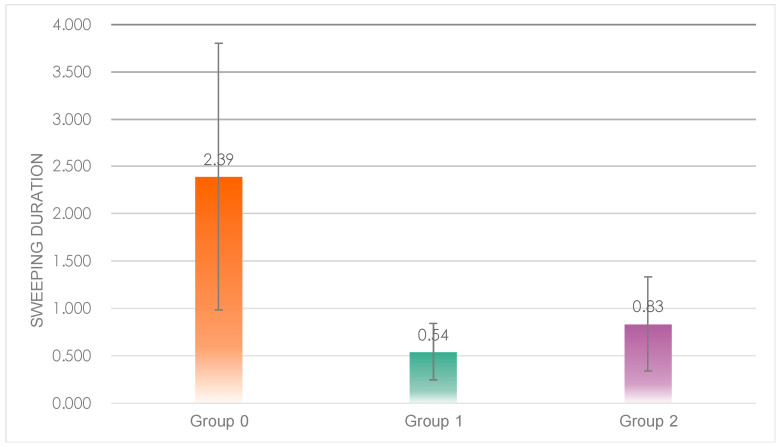
Individual differences in the total sweep duration of the three groups.

One-way ANOVA results showed significant differences in sweep time between the three groups (Figure 11), *F*(2, 20) = 7.245, *p* = 0.00 < 0.05; post hoc tests revealed that sweep time was significantly higher in Group 0 (*M* = 2.39, *SD* = 1.41) than in Group 1 (*M* = 0.54, *SD* = 0.30) and Group 2 (*M* = 0.83, *SD* = 0.50), with no significant difference between Group 0 and Group 1.

**Figure 12:**
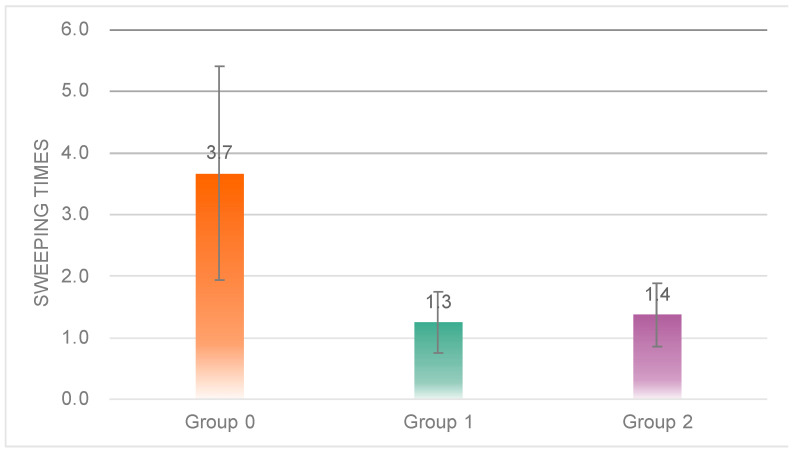
Individual differences in the total sweep times of the three groups.

One-way ANOVA results showed significant differences in the number of sweeps between the three groups (Figure 12), (*F*(2, 18) = 9.479, *p* = 0.00 < 0.05); post hoc tests revealed that the number of sweeps was significantly higher in Group 0 (*M* = 3.67, *SD* = 1.73) than in Group 1 (*M* = 1.25, *SD* = 0.50) and Group 2 (*M* = 1.38, *SD* = 0.52), with no significant difference between Group 0 and Group 1.

The results of the sweep data showed that subjects in Group 0 had significantly higher total sweep duration and times of sweeps than those in Group 1 and Group 2, indicating that the robot in Group 0 had a more engaging performance.

### 5.2. Experiment Two

#### 5.2.1. Safety

**Figure 13:**
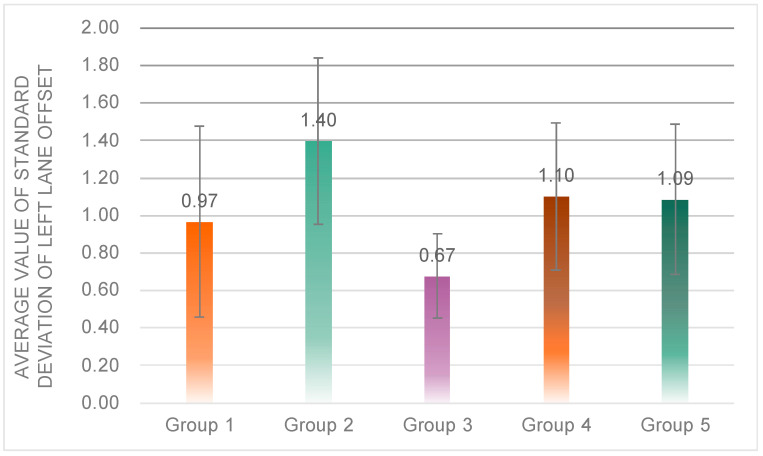
Average value of standard deviation of left lane offset of the five groups.

One-way ANOVA results showed that there was a significant difference in the average value of the standard deviation of left lane offset among the five groups (*F*(4, 72) = 6.507, *p* = 0.00 < 0.05) (Figure 13); post hoc tests revealed that the average value of the standard deviation of left lane offset of subjects in Group 3 (*M* = 0.67, *SD* = 0.22) was significantly lower than that in Group 1 (*M* = 0.97, *SD* = 0.51), Group 2 (*M* = 0.40, *SD* = 0.44), Group 4 (*M* = 1.10, *SD* = 0.40), and Group 5 (*M* = 1.09, *SD* = 0.40); the standard deviation of left lane offset of subjects in Group 2 was significantly higher than that in Group 1, 4, and 5.

In terms of safety, subjects in Group 3 had significantly better performance in lateral control of the vehicle and drove more safely. Further analysis leads to the following conclusions: (1) the result that the average value of the standard deviation of left lane offset of Group 1 is significantly lower than that of Group 2 indicates that the driver’s driving performance is better and the vehicle is safer when the robot’s expression is negative in this type of scenario; (2) the result of Group 3 with a significantly lower average value of the standard deviation of left lane offset than Group 1, Group 4 and Group 5 indicates that the robot’s speech speed at around 345 words/min maintains the safest driving.

#### 5.2.2. Workload

**Figure 14:**
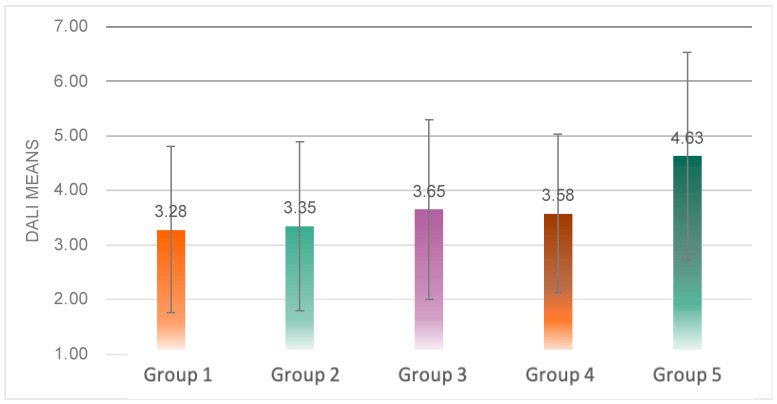
Average value of DALI score of the five groups.

Significant differences between groups were produced in the mean workload scores (*F*(4, 139) = 5.312, *p* = 0.001 < 0.05) (Figure 14). Multiple comparison analysis revealed that the average value of workload scores in Group 5 was significantly higher than in Group 1 (*p* = 0.003 < 0.05), Group 2 (*p* = 0.005 < 0.05) and Group 3 (*p* = 0.043 < 0.05).

**Figure 15:**
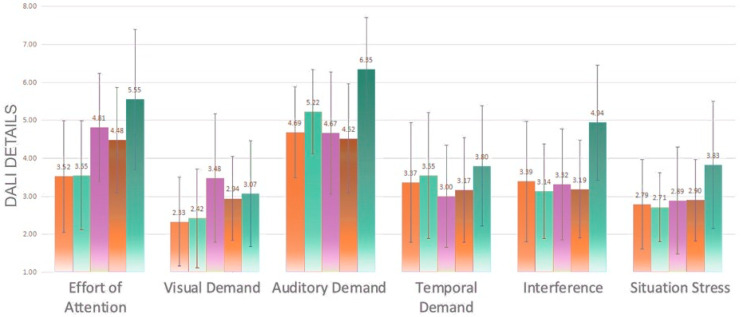
Detail values of the DALI scale of the five groups.

Significant differences in workload details arose between experimental groups on every dimension except time demands (Figure 15). Multiple comparison analysis revealed that Experimental Group 5 was significantly higher than Group 1 (*p* = 0.000 < 0.05) and Group 2 (*p* = 0.001 < 0.05) on the attention dimension; Group 3 was significantly higher than Group 1 (*p* = 0.042 < 0.05) on the visual demand dimension; Group 5 was significantly higher than Group 1 (*p* = 0.004 < 0.05), Group 3 (*p* = 0.002 < 0.05) and Group 4 (*p* = 0.000 < 0.05) on the auditory demand.

In terms of interference, Group 5 was significantly higher than all other experimental groups; in situational stress, dimension Group 5 was significantly higher than Group 2 (*p* = 0.040 < 0.05). This indicates that the speech rate of Group 5 makes the driver’s workload increase. The normal speech rate of Group 1 and Group 2 was able to reduce the workload of the subjects, mainly due to the reduction of attentional demands, especially visual demands.

#### 5.2.3. Usability

There was no significant difference between the groups (*F*(4, 135) = 1.377, *p* = 0.245). In the case of usability score details, there were also no significant differences above the three dimensions of satisfaction with difficulty (*F*(4, 126) = 2.350, *p* = 0.058), satisfaction with time spent (*F*(4, 121) = 0.053, *p* = 0.995), and satisfaction with help information (*F*(4, 124) = 1.324, *p* = 0.265).

#### 5.2.4. Trust

**Figure 16:**
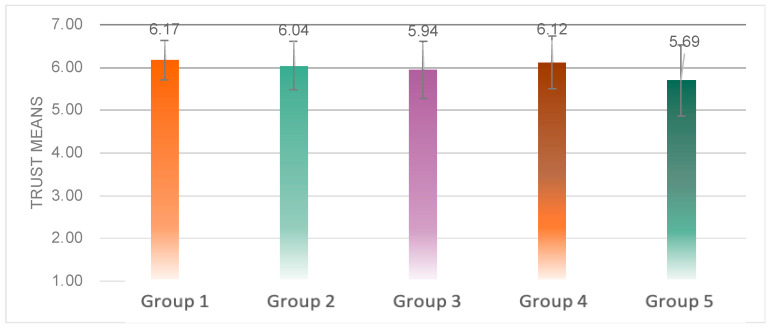
Average value of trust score of the five groups.

The average trust score was significantly different among the five groups (*F*(4, 99) = 3.744, *p* = 0.007 < 0.05) (Figure 16). The multiple comparison analysis determined that the trust score of Group 5 was significantly lower than that of Group 1 (*p* = 0.012 < 0.05), Group 2 (*p* = 0.045 < 0.05) and Group 4 (*p* = 0.017 < 0.05).

**Figure 17:**
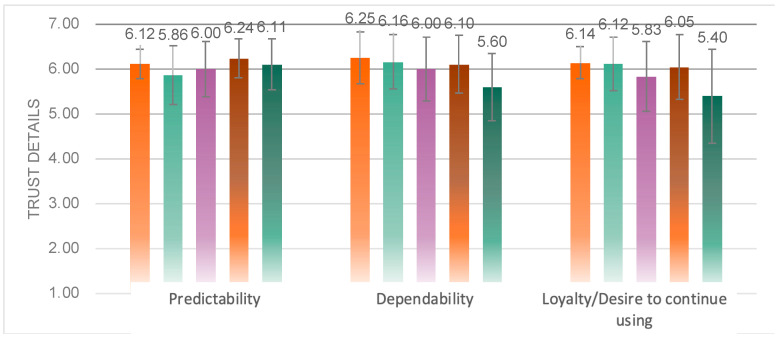
Detail values of the DALI scale of the five groups.

From the details of the trust scores (Figure 17), it was obtained that significant variability arose above the dimensions of dependability (*F*(4, 87) = 2.774, *p* = 0.032 < 0.05), desire to continue using (*F*(4, 86) = 3.099, *p* = 0.020 < 0.05), and not on the dimension of predictability (*F*(4, 86) = 1.315, *p* = 0.271) differences. Multiple comparison analysis yielded that Experimental Group 5 had a significantly lower dependability than Group 1 (*p* = 0.034 < 0.05), and Group 5 was significantly lower than Group 1 (*p* = 0.048 < 0.05) and Group 2 (*p* = 0.041 < 0.05) on the wish to continue using dimension.

#### 5.2.5. Sweeping

**Figure 18:**
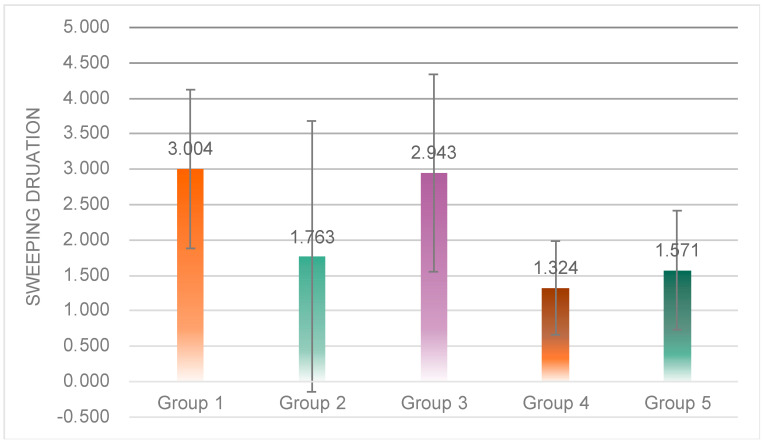
Individual differences in the total sweep duration of the five groups.

One-way ANOVA results showed significant differences in sweep time among the four experimental groups (*F*(4, 47) = 3.244, *p* = 0.02 < 0.05) (Figure 18). Post hoc tests revealed that the sweep time of subjects in Group 1 (*M* = 3.00, *SD* = 1.12) was significantly higher than that of subjects in Group 2 (*M* = 1.76, *SD* = 1.91), Group 4 (*M* = 1.32, *SD* = 0.66) and Group 5 (*M* = 1.57, *SD* = 0.84); subjects in Group 3 (*M* = 2.94, *SD* = 1.39) had significantly higher sweep times than Group 2, Group 4, and Group 5.

**Figure 19:**
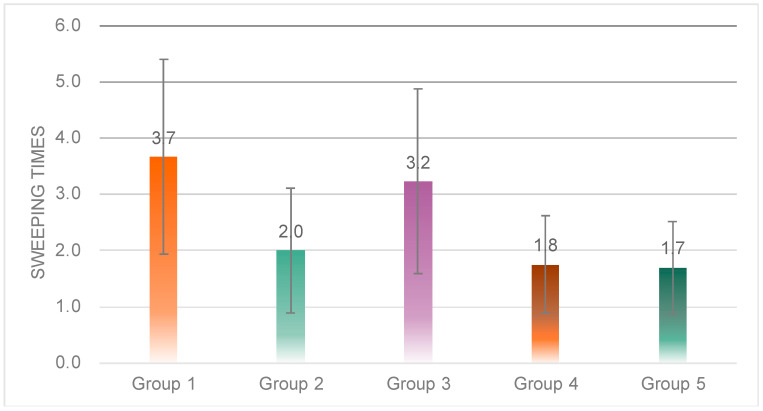
Individual differences in the total sweep times of the five groups.

One-way ANOVA results showed significant differences in the number of sweeps among the five groups (*F*(4, 53) = 2.931, *p* = 0.00 < 0.05) (Figure 19). Post hoc tests revealed that Group 3 (*M* = 3.23, *SD* = 1.64) was significantly higher than Group 2 (*M* = 2.00, *SD* = 1.11), Group 4 (*M* = 1.75, *SD* = 0.87) and Group 5 (*M* = 0.70, *SD* = 0.82); Group 1 (*M* = 3.37, *SD* = 1.73) was significantly higher than Group 2, 4 and 5.

The results of the sweep data show that the drivers in Group 3 had the highest sweep duration and number of sweeps to the robot, which indicates that the robot with this speech rate is most attractive for drivers to interact with. In terms of driving safety, it did not pose a threat to driving safety, as there were cases where the single sweep time exceeded 2s.

## 6. Discussion

While human-induced driving safety risks can be effectively reduced by robots assisting drivers in driving, the driving safety during human–robot interaction needs to be considered. It requires designing the robot’s expression to improve car control, trust, workload, and usability. This paper explores the application of the SAT model proposed by Chen et al. to the design of in-vehicle robot multimodal warnings while driving and explores the performance of the visual and auditory channels. This project aims to give optimal robot expression in driving safety warning scenarios.

The primary objectives were (1) to determine the influence of the robot’s multi-channel transparency patterns on driving safety and human outcomes; (2) to determine the effect of the robot’s speech rate and expression valence on driving safety and human outcomes.

### 6.1. Transparency

For driving safety warning scenarios, the most appropriate robot transparency pattern is to skip the SAT1-level stage, include visual and audio modalities in the SAT2-level stage, and include auditory modality in the SAT3-level stage. The visual modality should express facial expressions and the auditory modality should provide warning content. This transparency pattern provides the highest level of driver safety. 

The higher transparency for the in-vehicle robot can help the driver better understand the intent of the robot’s expression and reduce the driver’s need to reprocess the information. The results of this experiment also found that higher transparency means better usability. However, higher transparency does not necessarily provide better human performance when the driver’s attention resources are scarce, such as in scenarios where the driving task is the primary task.

In terms of visual modality, higher visual channel transparency exposes the driver to a higher workload, which may take up more cognitive resources and make it difficult for the driver to concentrate on driving. In addition, the visual channel was considered inappropriate to contain meaningful content. The results of Experimental Group 2 showed that the driver’s workload was not affected by changes in the robot’s expressions when the information related to the driver’s behavioral correction was not conveyed through the robot’s expressions. The visual channel was considered inappropriate to contain meaningful content.

In terms of auditory modality, the information in the voice can accurately convey the robot’s warning content, but the designer should pay attention to the accuracy of the expression and the conciseness of the text content. The voice in the SAT1-level stage affects the driver’s control of the vehicle. Therefore, the design should avoid adding modal particles or other information unrelated to the warning content. In this regard, previous studies have shown that robots were shown to reduce usability and increase workload because of the SAT1-level speech when the driver did not make a mistake, because SAT1-level anthropomorphic speech disturbed the driver while driving [50].

### 6.2. Speech Rate

Although the content of warnings requires speech to be delivered, designers cannot simply try to improve the efficiency of delivery by increasing the speed of speech. The vehicle control and workload showed the same trend when the speed of speech was varied. Thus, there is an optimal speech speed when the driver can properly discriminate the robot’s speech; slower or faster speech speeds will result in a lower driver workload and poorer vehicle control. When the robot provided warnings in Chinese speech, the appropriate speech rate was 345 words/minute, which is somewhat faster than the standard newscast speech rate. This should also be discussed in other different languages. Moreover, the DALI details indicate that appropriate speech speed improved driver attentional demands and visual demands as drivers better understood the robot’s speech.

In addition, the robot must avoid large changes in speech speed caused by system bugs, etc. Providing extremely fast speech to the driving driver would significantly increase the driver’s workload and affect car control. This extremely fast speech speed took up a significant amount of the driver’s attentional and auditory resources and caused a high level of interference. Combined with the findings above, this is not due to the driver’s attempt to understand the speech. Judging from the significant decrease in trust, it may be because the extremely fast speech reduces the anthropomorphism of the robot, thus surprising or confusing the driver.

### 6.3. Facial Expression

In the current speeding scenario, the negative expressions of the robot allowed the driver to drive better. Robots with negative expressions are more attractive to the driver, which enhances the communication between the driver and the robot, leading to better human–robot co-driving performance. This demonstrates that the negative bias phenomenon [57] also applies to the robot, making negative expressions more easily attract the driver’s attention and more easily discriminated by the driver, thus providing a better warning effect. This phenomenon did not affect driver safety by taking higher attentional resources. The effect of the two expression valences on human outcomes was almost the same except for sweeping. It may be that the two expressions have the same five senses, so they are the same in terms of anthropomorphism.

### 6.4. Sweeping

The driver’s ability to control the vehicle was positively correlated with how often and for how long they paid attention to the robot. When the robot made better performance, the driver was more willing to communicate with the robot, and smoother communication increased the driver’s confidence in the primary driving task, making the driver more mentally active.

## 7. Conclusions and Future Work

The current research applies a robot transparency model to the way intelligent robots provide multimodal warnings. When the in-vehicle robot has facial expressions and voice, the design of the robot’s multimodal warnings should follow the following guideline in order for the warnings to not increase the safety risk and to draw the driver’s attention to driving safety and the corrective operation to be performed sequentially through speech in the auditory modality. When informing the wrong operation, the robot should provide negative expressions; when informing the corrective operation, the robot avoids providing additional information on the visual modality. The Chinese speech rate should be 345 words/minute, which ensures safer driving when the driver receives warnings.

In conclusion, when the robot provides a warning, the content should be delivered to the driver via speech. The discourse should be organized to include an understanding of the robot driver’s current behavior and the expected behavior of the driver to change the status quo. The auditory channel should avoid the robot providing communicative information with the five senses of the warning content, but this does not mean that the process of human–robot communication should be reduced to mere information transfer and ignore the social element. In the visual channel, the robot’s facial expressions do not have obvious warning content, but reasonably designed expressions can improve driver–robot communication and improve driver–robot understanding, making the delivery of warning signals more effective and the delivery process safer. Additionally, whether it is critical information delivered through speech or non-critical information delivered through facial expressions, improving the driver’s attention level can help improve driving safety. More reasonable speech rates and negative facial expressions increase driver attention levels. This design guideline provides a reference for the interaction design of a driver assistance system with a robot as an interface, helping designers provide safer multimodal warnings. The research also informs robot speech and expressions when the driver drives dangerously. The current study also showed that driver safety is compromised when drivers have difficulty understanding robotic speech (e.g., very fast speech). Therefore, the design should avoid exaggerating the speech of the robot.

A limitation of this study is that the study of the “how” of the robot’s visual and auditory warnings did not extensively cover other variables of sound and vision that may affect the driver‘s driving performance. Future research can discuss the effects of psychoacoustic parameters, such as the pitch and volume of robot speech, and visual parameters, such as the style of the robot’s facial features. Research on other modalities such as haptics can also be further discussed. In addition, robot speech and facial expressions can also express emotions, so the impact of robot emotions on driving safety can continue to be explored.

## Data Availability

Data sharing is not applicable.
